# Spontaneous Variants of the [*RNQ*+] Prion in Yeast Demonstrate the Extensive Conformational Diversity Possible with Prion Proteins 

**DOI:** 10.1371/journal.pone.0079582

**Published:** 2013-10-25

**Authors:** Vincent J. Huang, Kevin C. Stein, Heather L. True

**Affiliations:** Department of Cell Biology and Physiology, Washington University School of Medicine, Saint Louis, Missouri, United States of America; Ohio State University, United States of America

## Abstract

Prion strains (or variants) are structurally distinct amyloid conformations arising from a single polypeptide sequence. The existence of prion strains has been well documented in mammalian prion diseases. In many cases, prion strains manifest as variation in disease progression and pathology, and in some cases, these prion strains also show distinct biochemical properties. Yet, the underlying basis of prion propagation and the extent of conformational possibilities available to amyloidogenic proteins remain largely undefined. Prion proteins in yeast that are also capable of maintaining multiple self-propagating structures have provided much insight into prion biology. Here, we explore the vast structural diversity of the yeast prion [*RNQ*+] in *Saccharomyces cerevisiae*. We screened for the formation of [*RNQ*+] *in vivo*, allowing us to calculate the rate of spontaneous formation as ~2.96x10^-6^, and successfully isolate several different [*RNQ*+] variants. Through a comprehensive set of biochemical and biological analyses, we show that these prion variants are indeed novel. No individual property or set of properties, including aggregate stability and size, was sufficient to explain the physical basis and range of prion variants and their resulting cellular phenotypes. Furthermore, all of the [*RNQ*+] variants that we isolated were able to facilitate the *de novo* formation of the yeast prion [*PSI*+], an epigenetic determinant of translation termination. This supports the hypothesis that [*RNQ*+] acts as a functional amyloid in regulating the formation of [*PSI*+] to produce phenotypic diversity within a yeast population and promote adaptation. Collectively, this work shows the broad spectrum of available amyloid conformations, and thereby expands the foundation for studying the complex factors that interact to regulate the propagation of distinct aggregate structures.

## Introduction

Protein misfolding disorders refer broadly to a class of human diseases associated with the failure of a protein or peptide to adopt its native, functional conformation [1]. Such misfolding can lead to the formation of fibrillar aggregates called amyloid. Amyloid fibers typically form as a β sheet-rich structure in a self-replicating process [2]. These highly ordered arrangements of β sheets are formed from non-covalent interactions of neighboring polypeptides in which the β strands run perpendicular to the fibril axis [1,3]. This fundamental architecture is shared among a variety of proteins associated with unrelated protein conformational disorders, including Alzheimer’s disease and Type II diabetes [4]. Interestingly, significant conformational variation can exist while still maintaining this generic amyloid structure [5]. Such amyloid polymorphism has been most studied in the context of prion strains, but recent data suggest that it is a common feature of many amyloidogenic proteins [6,7,8].

Prion diseases, also called transmissible spongiform encephalopathies (TSEs), represent a subset of protein misfolding disorders that are invariably fatal [9]. These diseases include bovine spongiform encephalopathy (BSE) in cattle and Creutzfeldt-Jakob disease (CJD) in humans. TSEs develop when the host-encoded prion protein, PrP^C^, assumes the abnormal β sheet-rich PrP^Sc^ conformation [10]. This infectious structure self-propagates by sequestering native PrP^C^ and templating further conversion to PrP^Sc^ [11,12].

Initial transmission experiments with PrP^Sc^ encountered what is now known as the “species barrier” [13]. This refers to the observation that transmission of PrP^Sc^ between two different species is typically far less efficient than transmission within the same species [14]. This barrier may be partially due to changes in amino acid sequence, but can also be due to changes in the self-propagating structure of the protein itself. Indeed, even within a single species, pathological variation in TSEs and different biochemical signatures of PrP^Sc^ have been observed, leading to the isolation of distinct PrP^Sc^ types [15,16]. These different types of PrP^Sc^ are called prion strains, and represent amyloid conformations of PrP that are structurally unique. In many cases, different prion strains show differences in biochemical properties, such as protease resistance or denaturant sensitivity, which correlate with variation in pathology and the time course of disease [17,18,19,20,21,22]. However, in other cases, prion strains have been isolated that vary in pathology, yet remain biochemically indistinguishable, according to the levels of sensitivity available with current assays [23]. Moreover, while genetic polymorphisms in PrP bias the formation of particular conformations of PrP^Sc^, a single primary sequence can propagate a multitude of distinct prion strains [14]. Indeed, it has been estimated that the range of heterogeneity seen in samples from patients with sporadic CJD represents over 30 distinct prion strains [24,25]. Clearly, the structural limits of amyloid polymorphism of prion strains are quite large.

Interestingly, functionally distinct prion proteins exist in fungi such as the yeast *Saccharomyces cerevisiae* [26,27]. Yeast prion proteins share many of the same misfolding and aggregation characteristics as the proteins associated with human protein conformational disorders. As such, yeast has provided a tractable model system to investigate many facets of protein aggregation and prion biology, including that of prion strain diversity. As in mammals, prion strains in yeast (termed prion variants) are conformationally distinct, self-propagating amyloid structures. This formation of amyloid in yeast leads to changes in cellular phenotypes, which typically resemble a loss-of-function phenotype of the prion protein [28,29,30,31]. One of the most well-studied prion proteins in *S. cerevisiae* is the translation termination factor Sup35. Sup35 is the eRF3 that normally exists in a complex that functions to recognize stop codons in mRNA and facilitate the release of polypeptide chains from ribosomes [32,33]. Conversion of Sup35 into its prion form, [*PSI*+], establishes a loss-of-function phenotype that is dominant and inherited in a non-Mendelian fashion [31]. In [*PSI*+] cells, much of the Sup35 is sequestered into prion aggregates, thereby impairing translation termination and causing readthrough of stop codons (also known as nonsense suppression) [34,35].

[*PSI*+] variants have been broadly classified into categories based on the degree of nonsense suppression [36]. Two well-characterized variants are strong [*PSI*+] and weak [*PSI*+]. Cells propagating the strong [*PSI*+] variant exhibit a greater amount of nonsense suppression as compared to cells propagating the weak [*PSI*+] variant [36]. Studies of strong and weak [*PSI*+] led to a model that proposed an explanation for how differences in the biochemical properties of these [*PSI*+] variants correlate with differences in biological phenotypes [5,37,38,39,40,41]. This model posits that decreased fiber stability results in increased fragmentation, thereby giving rise to a greater number of prion seeds, and thus more fibril "free ends" that can recruit and sequester natively-folded Sup35 [39]. Ultimately, the more “free ends” available are hypothesized to correlate to an increased rate of fiber growth that, in the case of the [*PSI*+] prion, modulates the strength of the nonsense suppression phenotype as the efficiency of translation termination is linked to the size of the soluble, active pool of Sup35 [41]. Interestingly, these trends have been recapitulated with some PrP^Sc^ strains, as lower aggregate stability correlated with a shorter incubation period and earlier onset of disease [42]. However, this correlation between aggregate stability and fiber growth does not explain differences in all PrP^Sc^ strains [21], or even in prion variants of another yeast prion, [*RNQ*+] [43]. Indeed, even with the [*PSI*+] prion, there may be multiple ways to acquire phenotypically similar prion variants [39,44,45]. Such differences highlight the remarkable conformational diversity of amyloid and the fact that there may be several ways to generate amyloid variant structures from a single polypeptide sequence.

 The [*RNQ*+] prion, comprised of the Rnq1 protein, is also called [*PIN*+] for [*PSI*+]-inducible, as it is required for the *de novo* formation of all [*PSI*+] variants *in vivo* [46,47,48,49,50]. It has been hypothesized that Rnq1 aggregates facilitate the formation of [*PSI*+] by interacting with soluble Sup35 and acting as an imperfect template to cross-seed the formation of [*PSI*+] [46]. Interestingly, a set of [*RNQ*+] variants was isolated that differ in their relative ability to influence the formation of [*PSI*+] [51]. Cells harboring some [*RNQ*+] variants afforded low levels of [*PSI*+] formation, while [*PSI*+] formed much more readily in cells propagating other [*RNQ*+] variants. Such cross-seeding between heterologous proteins may in fact be a common feature among protein conformational disorders [52]. Recently, variants of α-synuclein, the protein that misfolds and aggregates in Parkinson’s disease, have been reported to differentially influence the formation of tau inclusions [53]. However, what properties allow some amyloid structures to promote heterologous cross-seeding more efficiently than others remains unanswered.

Additional biochemical characterization of the [*RNQ*+] variants suggested that the variation in [*PSI*+] formation likely resulted from underlying structural differences between [*RNQ*+] variants and subsequent changes in the interaction with Sup35 [54,55,56]. However, as Rnq1 has no known function in its monomeric state, identification of spontaneously formed [*RNQ*+] cells has been cumbersome. As such, most of the [*RNQ*+] variants investigated to date were isolated by screening for [*PIN*+] cells (which turned out to be [*RNQ*+]) that had formed the [*PSI*+] prion [51]. Therefore, the conformational diversity observed in the set of previously characterized spontaneous [*RNQ*+] variants remains limited to aggregate structures of Rnq1 that are capable of inducing [*PSI*+].

 Here, we explore the degree of structural variation that is possible with the [*RNQ*+] prion when not linked to the ability to induce [*PSI*+]. We isolated a novel set of spontaneously formed [*RNQ*+] variants using a chimeric [*RNQ*+] reporter protein (RRP) that allowed us to phenotypically monitor the [*RNQ*+] status of a cell. The large set of [*RNQ*+] variants that we isolated exhibited a wide range of phenotypes, differing in terms of their [*RRP*+] phenotype, mitotic stability, aggregate size and stability, distribution pattern of aggregates *in vivo*, and in the ability to induce [*PSI*+]. We show that all of the [*RNQ*+] variants we obtained and analyzed in this study are distinct from those originally isolated [51]. These findings demonstrate the tremendous amount of conformational diversity that can be generated from a single amyloidogenic protein. By expanding the number of existing [*RNQ*+] variants, we are poised to better understand what factors dictate the ability of a given prion variant to form, propagate, and cause a particular phenotype. This will provide insight into the structural basis of prion strains and may help elucidate the mechanisms underlying pathological variation in protein misfolding diseases and the species transmission barrier of prion diseases.

## Materials and Methods

### Yeast Strains, Plasmids, and Media

All *S. cerevisiae* strains used in this study were derivatives of 74-D694 (*ade1-14 ura3-52 leu2-3,112 trp1-289 his3-Δ200*). Yeast harboring the s.d. low, s.d. medium, s.d. high, s.d. very high, or m.d. high [*RNQ*+] variants were kind gifts from Dr. Susan Liebman [51,54]. Construction of the yeast strains expressing RRP ([*RNQ*+] reporter protein) used to phenotypically monitor the [*RNQ+*] prion were previously described [57]. Briefly, by the pop-in/pop-out method, *SUP35* was replaced with the RRP gene encoding a fusion protein consisting of the prion-forming domain of Rnq1 and the M and C domains of Sup35. [*psi-*] [*RNQ*+] strains propagating the novel [*RNQ*+] variants described in this study were created by mating the [*RNQ*+] RRP strains to a wild-type 74-D694 [*psi*-] [*rnq*-] strain, and sporulating to obtain haploids containing *SUP35* instead of *RRP*. The presence of *SUP35* and the absence of *RRP* were confirmed by colony PCR and western blot.

pEMBL-*SUP35* was created through a similar process as a previously described pEMBL-*SUP2* plasmid [58]. The *SUP35* promoter was first amplified using oligonucleotides 5'-CGCCTCGAGGACGACGCGTCACAGTG and 5'-CCCGGATCCTGTTGCTAGTGGGCAGATATAG, digested with XhoI/BamHI, and ligated into pEMBL-yex4 (2μ, *URA3*). The *SUP35* open reading frame and terminator were then amplified using oligonucleotides 5'-CGCGGATCCACTAGTATGTCGGATTCAAACCAAGG and 5’-GGGGAGCTCGTGATTGAAGGAGTTGAAACCTTGC, digested with BamHI/XbaI, and ligated, thereby disrupting the GAL-CYC1 promoter of pEMBL-yex4. 

Yeast were grown at 30°C in YEPD (complete media containing 2% dextrose, 2% peptone, 1% yeast extract) or synthetic dextrose (SD) media (2% dextrose, 0.67% yeast nitrogen base without amino acids) lacking the indicated nutrients to select for transformed plasmids, [*PSI*+] cells, or [*RNQ*+] RRP cells. As indicated, ¼YEPD plates containing 0.25% yeast extract were used to better visualize colony color phenotypes. Transient growth on YEPD containing 3mM guanidine hydrochloride (GdnHCl) was used to check curability of the [*PRION*+] status, as GdnHCl inhibits the chaperone Hsp104 that is required for prion propagation [59,60,61].

### Prion Color Assay

The yeast strain 74-D694 harbors the *ade1-14* allele having a premature nonsense mutation that can be used to easily monitor the [*PSI*+] status of cells [62]. Soluble Sup35 in [*psi*-] cells functions to faithfully terminate translation at the premature stop codon. As such, these cells are unable to complete the adenine biosynthetic pathway, cannot grow on medium lacking adenine, and appear red when grown on a rich medium, such as YEPD, due to the accumulation of a metabolic intermediate in the pathway. Conversely, the aggregation of Sup35 in [*PSI*+] cells results in readthrough of the nonsense mutation in *ade1-14*, thereby allowing cells to grow on medium lacking adenine. The extent of nonsense suppression can vary depending on the prion variant that propagates and the associated degree of Sup35 sequestration. When Sup35 is efficiently sequestered in strong [*PSI*+] cells, colonies are white and grow robustly on SD-ade. In contrast, weak [*PSI*+] cells do not sequester Sup35 as efficiently and are pink in color on YEPD and grow less well on SD-ade. As described previously, RRP can functionally replace Sup35. Analogous to Sup35, RRP co-aggregates with Rnq1 in [*RNQ*+] cells, but remains soluble and is functional in translation termination in [*rnq*-] cells [57]. The RRP construct in 74-D694 allows [*RNQ*+] variants to be distinguished phenotypically in the same manner as [*PSI*+] variants. Before evaluating color phenotypes, YEPD plates were moved from 30°C to 4°C for at least one day to allow the colony color to develop.

### Spontaneous [RNQ+] Formation

The spontaneous formation of [*RNQ*+] was quantified using a [*rnq*-] strain expressing RRP and following previously described methods with some modifications [57,63]. This strain was transformed with pRS415-*ura3-197*, a kind gift from Dr. Yoshikazu Nakamura, that carries an allele of *URA3* with a nonsense mutation (UGA) at W197, which is suppressed in [*PSI*+] and [*RNQ*+] RRP cells [64]. Eight single transformants were independently grown in SD-leu to an OD_600_ of ~1.6. 150μl of each culture was plated onto SD-ade-ura medium to select for colonies that could suppress both the *ade1-14* and *ura3-197* alleles. These plates were incubated at 30°C overnight, moved to 4°C for two weeks (as cold has been shown to enhance *de novo* prion formation [46]), and then incubated at 30°C for another two weeks. All colonies that had acquired the white or pink phenotype indicative of *ade1-14* and *ura3-197* suppression were counted and spotted onto YEPD, YEPD containing 3mM GdnHCl, and SD-ade. Colonies were scored as true [*RNQ+*] if growth on YEPD+3mM GdnHCl resulted in a permanent color change from white or pink to red, thereby demonstrating curability of the [*RRP*+] phenotype. 

The total number of cells plated on the SD-ade-ura medium for each independent culture was determined by plating 200μl of a 1:10,000 dilution of the overnight culture onto SD-leu, which selected for the presence of pRS415-*ura3-197*. The total number of cells plated on SD-ade-ura for each culture was calculated by multiplying the number of colonies counted on SD-leu by a dilution factor of 52,500, calculated as follows: (# of colonies on SD-leu/200μl) * 10,000 * 150μl * 7 SD-ade-ura plates.

### Mitotic Stability of [RNQ+]

The mitotic stability of [*RNQ*+] was determined as previously described [43]. Independent colonies of each [*RNQ*+] variant expressing RRP were grown overnight in YEPD to an OD_600_ of ~2.5. 250μl of a 1:10,000 dilution of each culture was plated onto 13cm diameter YEPD plates. Mitotic loss of the [*RNQ+*] prion was scored as any red colony or any colony exhibiting red sectoring. Over 1,500 colonies were counted for each [*RNQ*+] variant. The percentage of mitotic loss was calculated by dividing the number of red and red sectoring cells by the total number of cells. [*RNQ*+] variants were characterized as mitotically stable if the percentage of prion loss was less than 0.5%. Additionally, cells were grown in YEPD medium overnight and normalized by OD_600_, followed by spotting 5-fold serial dilutions of cells onto ¼YEPD, YEPD+3mM GdnHCl, and SD-ade plates. Plates were incubated at 30°C for 3 days for the rich media and 7 days for SD-ade.

### Thermal Stability Assay

To analyze the stability of Rnq1 aggregates, cells were washed and lysed with 425-600μm acid-washed glass beads (Sigma-Aldrich) by vortexing in buffer containing 100mM Tris-HCl pH 7.5, 200mM NaCl, 1mM ethylenediaminetetraacetic acid (EDTA), 5% glycerol, 0.5mM dithiothreitol (DTT), 50mM N-ethylmaleimide (NEM), 3mM phenylmethylsulfonyl fluoride (PMSF), and Roche complete mini protease inhibitor cocktail for 3 min at high speed at 4°C twice with a 5 min incubation on ice in between. After adding an equal volume of RIPA buffer (50mM Tris-HCl pH 7.0, 200mM NaCl, 1% Triton X-100, 0.5% sodium deoxycholate, 0.1% SDS) following lysis, cell debris was removed by centrifugation at 3,300g for 15 seconds. Pre-cleared lysates were incubated in sample buffer (50mM Tris-HCl pH 6.8, 10% glycerol, 2% SDS, 100mM DTT) and treated for 5 minutes across a temperature gradient ranging between 45-95°C, and also at 25°C and 100°C. Samples were analyzed by SDS-PAGE. When subjected to higher temperatures, Rnq1 aggregates are solubilized and able to enter the SDS-PAGE gel. Protein separated by SDS-PAGE was then transferred to PVDF membrane for western blotting with an anti-Rnq1 antibody. ImageJ was used to quantify the resulting bands. All readings were normalized to the 100°C band, and the results were plotted using Origin 8.1 software. 

### Semi-Denaturing Detergent Agarose Gel Electrophoresis

SDD-AGE was performed as previously described with slight modifications [57]. Yeast cells were lysed by vortexing with glass beads as described above in buffer containing 25mM Tris-HCl pH 7.5, 100mM NaCl, 1mM EDTA, Roche complete mini protease inhibitor, 0.5mM DTT, 3mM PMSF, 5μg/mL pepstatin, and 40mM NEM. Cell debris was cleared by centrifugation at 3,300g for 30 sec. Lysates were incubated with sample buffer (60mM Tris-HCl pH 6.8, 5% glycerol, 2% SDS) for 7 minutes at room temperature and 40μg of protein was separated on a 1.5% Tris-glycine agarose gel. Protein was transferred to PVDF membrane overnight and analyzed by western blot using an anti-Rnq1 antibody.

### [*PSI*+] Induction

The *de novo* formation of [*PSI*+] was monitored as previously described [43,57]. Yeast propagating different [*RNQ*+] prion variants were transformed with pEMBL-*SUP35*. At least three independent overnight cultures were started for each prion variant in SD-ura and grown to an OD_600_ of 0.6-1.5. Each culture was diluted roughly 1:8,000 in water before plating 250μl onto 13cm diameter ¼YEPD plates and incubated at 30°C for 5 days, followed by overnight incubation at 4°C for color development. [*PSI*+] colonies were scored as any white/pink colonies or colonies having white/pink sectoring. More than 1,200 colonies were scored for each [*RNQ*+] variant. Previous studies have reported that only ~12% of the white/pink sectoring colonies are the result of nonheritable amyloids that cause Sup35 over-expression-dependent nonsense suppression, while the rest contain *bona fide* [PSI+] [57,65]. As an alternative means of assessing [*PSI*+] induction, overnight cultures were normalized by OD_600_ and spotted in 5-fold serial dilutions onto ¼YEPD, YEPD+3mM GdnHCl, and SD-ade. Plates were incubated at 30°C for 3 days for the rich media and 9 days for SD-ade.

To characterize the [*PSI*+] variants that formed, ~300 individual [*PSI*+] colonies of each [*RNQ*+] variant were spotted onto ¼YEPD, YEPD+3mM GdnHCl, and SD-ade. Plates were incubated at 30°C for 3 days for the rich media and 6 days for SD-ade. Based primarily on growth on SD-ade, [*PSI*+] variants that were confirmed curable after transient growth on YEPD+3mM GdnHCl were classified as one of the following: very weak (≤3 colonies in the spot), weak (growth covering up to 50% of the spot), medium (growth covering 50-90% of the spot), or strong (dense growth covering >90% of the spot in addition to white colonies present on ¼YEPD). 

### Microscopy

When expressed in [*RNQ*+] cells, Rnq1-GFP decorates Rnq1 aggregates to form fluorescent foci in prion variant-specific patterns, while remaining diffuse in [*rnq*-] cells [48,55]. Cells were transformed with the copper inducible pRS316*CUP1*-*RNQ1*(*153-405*)*-GFP*, a kind gift from Dr. Susan Lindquist, as used in previous studies [48,55]. Overnight cultures were grown to an OD_600_ of ~1.0 in SD-ura before backdiluting to an OD_600_ of 0.2 in SD-ura containing 50μM CuSO_4_ for ~2.5 hours prior to imaging. Samples were prepared on agar pads (3% wt/vol in liquid SD-ura) and plated directly onto VWR 3in x 1in, 1mm thick microscope slides. Fisherbrand No.#1.5 microscope coverslips were secured with nail polish. Images were collected using a Zeiss Axiovert 200 Inverted Microscope equipped with a Zeiss 100x/1.4 NA oil objective. Slidebook 5.0 (Intelligent Imaging Innovations) was used to analyze captured images and deconvolve (no neighbors algorithm) GFP Z-stacks.

## Results

### Rate of spontaneous [*RN*Q+] formation

In analyzing the conformational diversity of the [*RNQ*+] prion, we first wanted to quantify the rate of spontaneous [*RNQ*+] formation and compare it to what was previously described for [*PSI*+] and other yeast prions. As the [*RNQ*+] prion has no easily observable phenotype other than its essential role in [*PSI*+] induction, we used a chimeric protein called RRP ([*RNQ*+] reporter protein), which we created previously to monitor the [*RNQ*+] status of cells [57]. RRP consists of the prion-forming domain (PFD) of Rnq1 (amino acids 153-405) fused to the M and C domains of Sup35 (amino acids 124-685), such that the fusion retains functionality in translation termination. In its soluble form, RRP acts as the functional equivalent to Sup35 by faithfully recognizing stop codons to terminate translation. However, in [*RNQ*+] cells, RRP and Rnq1 co-aggregate to cause global [*PSI*+]-like nonsense suppression. 

To screen for the spontaneous formation of [*RNQ*+], we used a 74-D694 [*rnq*-] strain with the endogenous *SUP35* gene replaced by *RRP*. This strain harbored the [*PSI*+] and [*RRP*+] suppressible *ade1-14* and *ura3-197* alleles. As such, to monitor conversion to [*RNQ*+], we plated overnight cultures onto SD-ade-ura medium to select for cells that could suppress the premature stop codons in both alleles in a [*RRP*+]-dependent manner. Cells were scored as [*RNQ*+] if phenotypic curability was demonstrated after growth on medium containing guanidine hydrochloride (YEPD+3mM GdnHCl), which inhibits the chaperone Hsp104 required to propagate yeast prions [59,60,61]. Sedimentation assays were used to confirm the [*RNQ*+] status of a subset of these colonies by the presence of Rnq1 in the insoluble fraction (data not shown). Of the ~9.7x10^7^ cells plated on SD-ade-ura and >2,600 colonies spotted, 288 [*RNQ*+] colonies were identified (Table 1). This gave a rate of ~2.96x10^-6^ for the spontaneous formation of [*RNQ*+], which is slightly higher than the rate of [*PSI*+] formation previously reported [63].

**Table 1:** Rate of spontaneous [*RNQ*+] formation.

	**Replicate**	**# [*RRP*+] Colonies**	**Colonies on SD-leu**	**Total Colonies Plated on SD-ade-ura (x10^6^)**	**Rate of Spontaneous [*RNQ*+] Formation (x10^-6^)**
	1	54	136	7.14	7.56
	2	33	265	13.91	2.37
	3	34	355	18.64	1.82
	4	41	350	18.38	2.23
	5	34	164	8.61	3.95
	6	33	202	10.61	3.11
	7	26	212	11.13	2.34
	8	33	168	8.82	3.74
**Total**		288	1852	97.23	2.96

Eight independent overnight cultures of [*rnq*-] cells (*ura3-197*, *ade1-14*) expressing RRP were plated onto SD-ade-ura to select for nonsense suppressors, and to SD-leu to calculate the total number of colonies plated. [*RNQ*+] colonies were identified by curability after transient growth on YEPD+3mM GdnHCl.

### Spontaneous [*RN*Q+] formation yields mixed population of [*RN*Q+] variants

Interestingly, the spontaneously formed [*RNQ*+] colonies showed differences in growth on SD-ade, indicative of varying levels of nonsense suppression (data not shown). As the degree of nonsense suppression is a measure of distinct prion variants of [*PSI*+] [36] and [*RNQ*+] using RRP [43], this suggested that multiple [*RNQ*+] variants had formed spontaneously in our screen. 

**Figure 1:**
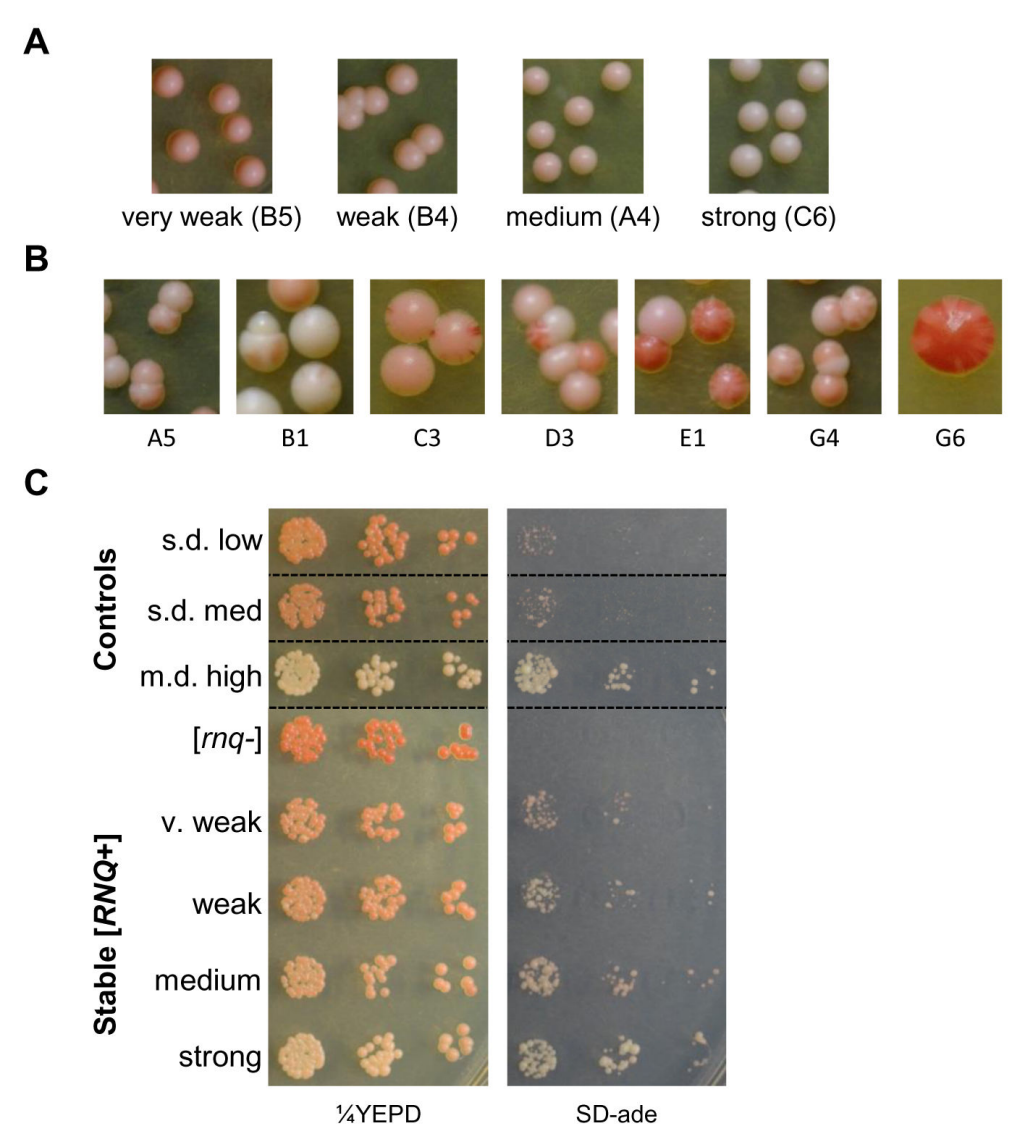
Spontaneous [*RNQ*+] formation produces multiple variants with unique [*RRP*+] phenotypes. Cultures of cells expressing RRP and harboring a spontaneous [*RNQ*+] variant were plated onto YEPD and scored for color and sectoring phenotypes. [*RNQ*+] variants were characterized as (A) stable or (B) unstable. Representative pictures are shown for each variant. (C) Normalized numbers of cells expressing RRP and harboring the indicated [*RNQ*+] variant were plated in 5-fold serial dilutions onto ¼YEPD and SD-ade media. Dotted lines denote where spots from the same plate have been cropped for clarity.

We wanted to isolate a subset of conformationally distinct [*RNQ*+] variants in order to perform a more extensive analysis on what types of aggregate structures were formed. Previous studies have shown that one property that can differ with variants of [*PSI*+] and [*RNQ*+] is the frequency of loss during mitotic division [36,43]. Therefore, to examine mitotic stability, overnight cultures of yeast harboring a large subset of our spontaneous [*RNQ*+] variants expressing RRP were plated onto YEPD. This produced a wide range of colorimetric phenotypes that were initially categorized as either stable or unstable. Stable [*RNQ*+] variants exhibited colonies that had a homogenous [*RRP*+] phenotype of one colony color (Figure 1A). Unstable [*RNQ*+] variants, on the other hand, displayed heterogeneous [*RRP*+] phenotypes, often within a single colony (Figure 1B). Some of these unstable [*RNQ*+] variants displayed a high frequency of mitotic loss, as indicated by the presence of red sectors or colonies that were entirely red. Other unstable [*RNQ*+] variants showed a mix of non-red colonies, including colonies that exhibited sectors of various shades of pink, and appeared similar to previously reported yeast strains harboring “undifferentiated” and “unspecified” variants of [PSI+] [66,67].

Four [*RNQ*+] variants were selected that had a stable colony color phenotype with little mitotic loss (<0.25%). These mitotically stable variants differed in the degree of [*RRP*+]-mediated nonsense suppression, and are referred to hereafter as strong [*RNQ*+] (white colonies), medium [*RNQ*+] (light pink), weak [*RNQ*+] (medium pink), and very weak [*RNQ*+] (dark pink). This classification helps distinguish our [*RNQ*+] variants from the [*RNQ*+] variants previously described, in which the nomenclature corresponded to different levels of [*PSI*+] induction and the pattern of Rnq1-GFP fluorescence [51,55]. To compare these two sets of [*RNQ*+] variants phenotypically, we spotted serial dilutions of normalized cells onto ¼YEPD and SD-ade plates (Figure 1C). Relative growth on SD-ade showed that very weak [*RNQ*+], weak [*RNQ*+], and medium [*RNQ*+] displayed intermediate growth between that of s.d. medium [*RNQ*+] and m.d. high [*RNQ*+], whereas strong [*RNQ*+] had more robust [*RRP*+]-mediated nonsense suppression and growth that was comparable to that of m.d. high [*RNQ*+].

Seven mitotically unstable [*RNQ*+] variants were also chosen for further analysis. C3 and G6 exhibited high frequencies of mitotic loss, while A5, B1, D3, E1, and G4 displayed a heterogeneous mixture of non-red sectoring [*RRP*+] phenotypes. For instance, many E1 colonies were dark pink with light pink sectors, while many B1 colonies were white with light pink sectors. These phenotypes can be contrasted with true mitotic loss, which is represented by sectoring to red. Interestingly, upon subsequent restreaking, the sectoring phenotypes of A5 and C3 stabilized into homogenous populations of colonies (data not shown). 

### [*RR*P+] phenotype does not correlate with [*PS*I+] induction

As the original [*RNQ*+] variants were classified by their ability to template the formation of [*PSI*+], we wanted to determine the efficiency with which our spontaneously formed [*RNQ*+] variants facilitated conversion to [*PSI*+]. We began by generating [*psi*-] [*RNQ*+] strains with each of our [*RNQ*+] variants. We obtained haploids expressing *SUP35* in place of *RRP* by mating the RRP-expressing strains to [*psi*-] [*rnq*-] cells to obtain diploids, followed by random sporulation or tetrad dissection and selecting haploids of the appropriate genotype. Interestingly, we noticed that there were two distinct populations of [*RNQ*+] RRP B1 colonies with contrasting [*RRP*+] phenotypes, one white with extensive light pink sectors (B1 P/W) and the other predominately white with very minor pink sectoring (B1 W). We proceeded with the independent mating of cells having each of these phenotypes to determine whether differences in [*RRP*+] phenotypes of clones from the same population would be reflected in [*PSI*+] induction efficiency. Both the diploids and haploids we obtained following sporulation were red in colony color, as these [*psi-*] [*RNQ*+] cells now had soluble wild-type Sup35 to function in faithful translation termination.

**Figure 2:**
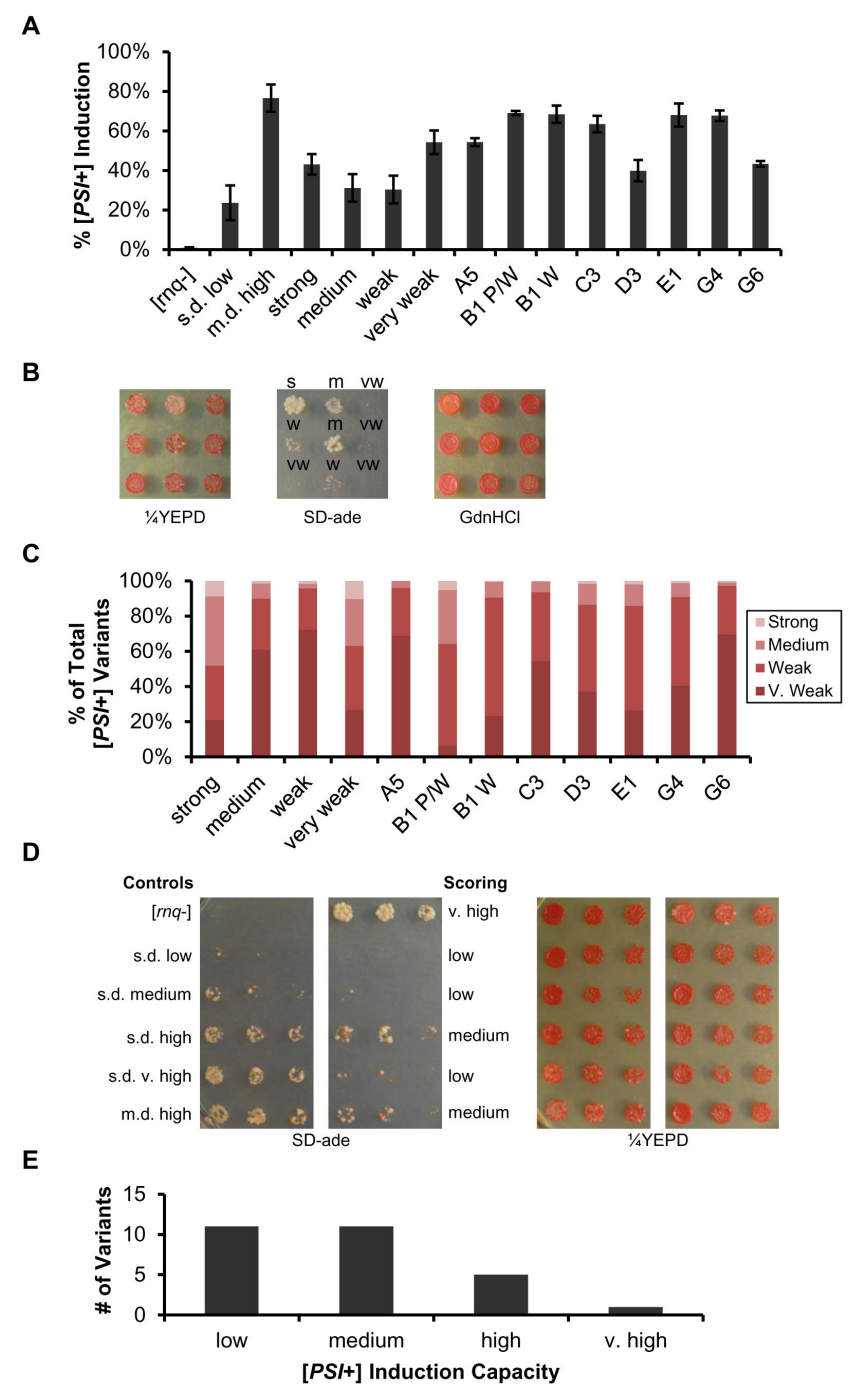
The [*PSI*+] induction rate and [*PSI*+] variant distribution is variable. (A) The induction of [*PSI*+] by over-expressing Sup35 in our 12 [*RNQ*+] variants was analyzed quantitatively by counting the number of [*PSI*+] colonies that formed on ¼YEPD plates from at least three independent experiments. Error bars represent standard error. Nonsense suppression by nonheritable amyloid due to the over-expression of Sup35 has been previously shown to only account for a small percentage of white/pink sectoring colonies (B) Example of scoring the newly induced [*PSI*+] variants as very weak (vw), weak (w), medium (m), or strong (s) by spotting [*PSI*+] colonies onto ¼YEPD, YEPD+3mM GdnHCl, and SD-ade media. (C) Counts of [*PSI*+] variants were plotted as a fraction of total [*PSI*+] colonies scored. (D) Example plate of scoring the ability of spontaneous [*RNQ*+] variants to induce [*PSI*+] upon Sup35 over-expression. [*PSI*+] induction efficiency was scored into one of four classifications by assessing colony growth on SD-ade relative to that of five previously described [*RNQ*+] variants: low (ranges from no growth to less growth than s.d. medium), medium (growth equivalent to s.d. medium to less than s.d. high), high (growth equivalent to s.d. high to less than s.d. very high), and very high (growth equivalent to s.d. very high or greater). (E) The types of [*RNQ*+] variants that spontaneously formed categorized based on the levels of [*PSI*+] induction.

In order to assess how well each [*RNQ*+] variant could facilitate [*PSI*+] induction, we transformed the progeny we generated with a plasmid over-expressing Sup35. After overnight growth in selection medium to maintain expression of the transformed plasmid, cells were plated onto ¼YEPD plates. Any pink/white colonies or colonies with pink/white sectoring were scored as [*PSI*+], and the rate of [*PSI*+] formation was calculated as the number of those colonies divided by the total number of cells plated (Figure 2A). For comparison, we also confirmed the rates of [*PSI*+] formation for the previously published [*RNQ*+] variants, along with [*rnq*-] cells.

 Confirming our previous results [43], the efficiency of [*PSI*+] induction for the previously described s.d. low, s.d. medium, and m.d. high [*RNQ*+] variants positively correlated with their [*RRP*+] phenotypes. Higher [*PSI*+] induction corresponded to a stronger [*RRP*+] phenotype. However, as we previously observed with [*RNQ*+] variants formed from transforming *in vitro* fibers [43], this trend did not hold for our set of stable [*RNQ*+] variants. Strong [*RNQ*+] and very weak [*RNQ*+] induced [*PSI*+] at fairly similar frequencies (45% and 54% respectively), while medium [*RNQ*+] and weak [*RNQ*+] exhibited lower rates of [*PSI*+] induction comparable to that of s.d. low [*RNQ*+]. Interestingly, many of our unstable [*RNQ*+] variants induced the formation of [*PSI*+] at higher rates relative to our stable [*RNQ*+] variants. Six of the eight unstable [*RNQ*+] variants induced [*PSI*+] very efficiently, at rates between 50-70%, while D3 and G6 facilitated [*PSI*+] induction at slightly lower rates of ~40%.

 Knowing that different [*RNQ*+] variants can promote the formation of [*PSI*+] to different extents, we were interested in determining whether the distinct amyloid structures of our [*RNQ*+] variants would bias the formation of particular [*PSI*+] variants, as reported in studies with other [*RNQ*+] variants [55,56]. To quantitatively evaluate the [*PSI*+] variant profile that corresponded to each of our [*RNQ*+] variants, we spotted newly induced [*PSI*+] colonies onto a set of ¼YEPD, YEPD+3mM GdnHCl, and SD-ade plates. Growth on SD-ade after six days of incubation at 30°C was used as the primary determinant in scoring the strength of each [*PSI*+] variant as very weak, weak, medium, or strong (Figure 2B-C). All 12 [*RNQ*+] variants appeared capable of inducing multiple variants of [*PSI*+], but the vast majority of the [*PSI*+] variants were categorized as very weak [*PSI*+] or weak [*PSI*+]. Indeed, A5 induced no strong variants of [*PSI*+]. In contrast, strong [*RNQ*+], very weak [*RNQ*+], and B1 P/W induced noticeably stronger [*PSI*+] variants than the other [*RNQ*+] variants in the subset. Interestingly, while B1 P/W and B1 W supported similar rates of [*PSI*+] formation, B1 P/W induced markedly stronger variants of [*PSI*+] than B1 W. Finally, as with the rate of [*PSI*+] induction, no correlation appeared to exist between the [*RRP*+]-mediated nonsense suppression phenotype of the four stable [*RNQ*+] variants and their corresponding induced [*PSI*+] variant profile.

 To further investigate the extent to which our spontaneously formed [*RNQ*+] variants can induce conversion to [*PSI*+], we generated 28 more [*psi*-] [*RNQ*+] haploids expressing *SUP35* in place of *RRP* as described above. These yeast strains propagated other [*RNQ*+] variants that had spontaneously formed in our initial screen. To monitor [*PSI*+] induction capacity, we over-expressed Sup35 as above and spotted normalized numbers of cells onto ¼YEPD and SD-ade plates (Figure 2D). As the overall growth of [*PSI*+] colonies on SD-ade is presumably influenced by both the rate of [*PSI*+] induction and the strength of the [*PSI*+] variant induced, we used this as a qualitative measure of [*PSI*+] induction efficiency by comparing the growth on SD-ade to that of the five previously published [*RNQ*+] variants and a [*rnq*-] control strain. Surprisingly, even though we screened for [*RNQ*+] formation independent of the ability to form [*PSI*+], all 28 spontaneously formed [*RNQ*+] variants were able to induce [*PSI*+] to some degree. By comparing our [*RNQ*+] variants to those previously published, we further scored the [*PSI*+] induction capacity of our [*RNQ*+] variants as low, medium, high, or very high (Figure 2E). Interestingly, an overwhelming majority of our [*RNQ*+] variants (22 out of 28) were categorized as inducing [*PSI*+] at low or medium levels. We identified E5 as an outlier in the data set in displaying robust growth on SD-ade, even greater than that of s.d. very high [*RNQ*+].

### Rnq1-GFP aggregation patterns of [*RN*Q+] variants

In addition to [*PSI*+] induction levels, fluorescence microscopy was also utilized to identify the original [*RNQ*+] variants as potentially distinct structures [55]. With expression of an inducible Rnq1(153-405)-GFP fusion construct [48,55], [*rnq*-] cells were shown to display diffuse fluorescence. In [*RNQ*+] cells, however, the fluorescent signal was localized in discrete, cytosolic foci. Two distinct GFP aggregation patterns emerged among the [*RNQ*+] variants: single-dot (s.d.), in which cells had one focus of GFP fluorescence, or multiple-dot (m.d.) where cells had multiple foci. As such, we set out to examine the Rnq1(153-405)-GFP fluorescence characteristics of 12 of the [*RNQ*+] variants that we had isolated.

**Figure 3:**
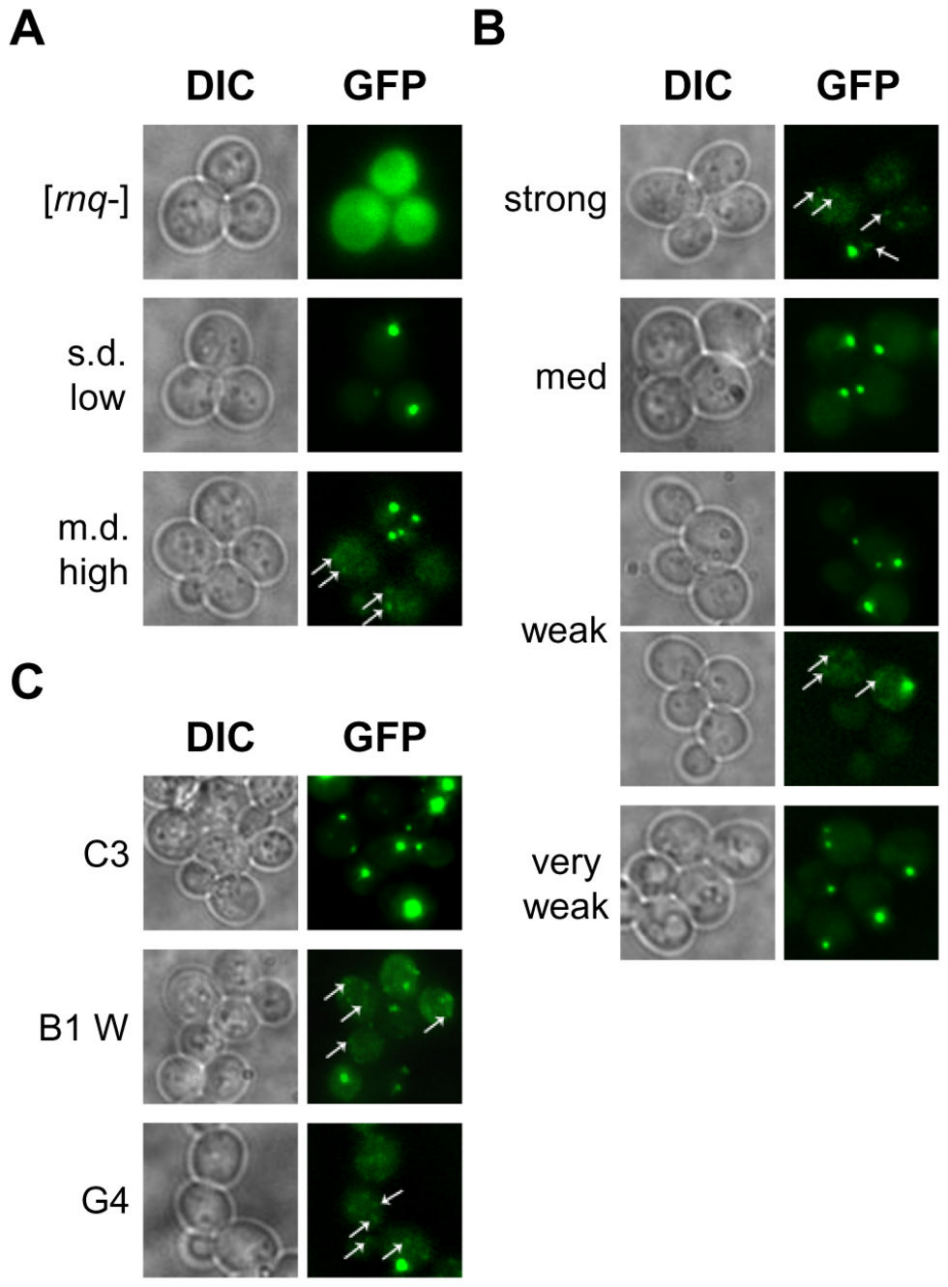
Spontaneously formed [*RNQ*+] variants show diversity in Rnq1-GFP aggregation patterns. Cells were transformed with a plasmid expressing Rnq1(153-405)-GFP under the *CUP1* promoter. Prior to imaging, transformants were transferred to induction medium containing 50μM CuSO_4_ and grown for ~2.5 hours. A variety of fluorescence patterns were observed, including: a single focus, large multiple foci, and numerous petite foci. Arrows identify petite foci that are fainter and smaller. Representative images in differential interference contrast mode (DIC) and under a GFP-emitted light filter (GFP) are shown for (A) previously published [*RNQ*+] variants and [*rnq*-], (B) all of the stable [*RNQ*+] variants, and (C) a subset of the unstable [*RNQ*+] variants.

 Previous studies have reported that, in their respective yeast strains, both the s.d. and m.d. Rnq1-GFP fluorescence patterns form with great efficiency, appearing in >75% of s.d [*RNQ*+] cells or >50% of m.d. [*RNQ*+] cells, respectively [55,68]. We recapitulated these findings for s.d. low [*RNQ*+], but also found that m.d. high [*RNQ*+] displayed a third Rnq1-GFP pattern as the dominant population (Figure 3A). This pattern was characterized by many petite foci (p.f.) that were much smaller and fainter in intensity than those observed with the s.d. and m.d. patterns.

 When analyzing the Rnq1-GFP aggregation patterns of our stable and unstable [*RNQ*+] variants, we found that these variants often did not fall into a strict bimodal classification of s.d. or m.d. (Figure 3B-C). Instead, we observed a gradient of patterns, which varied in the relative proportions of s.d., m.d., and p.f. cells (Table 2). Placed at one end of the spectrum, s.d. low [*RNQ*+] and our medium [*RNQ*+] had ~85-95% of foci-containing cells having the s.d. pattern. Although the majority of very weak [*RNQ*+], A5, and C3 cells similarly had the s.d. pattern, these variants also had an appreciable number of cells (~15-25%) that harbored the m.d. fluorescence pattern. In contrast, at the other end of the spectrum, the Rnq1-GFP aggregate pattern of m.d. high [*RNQ*+] consisted of both p.f. and m.d. patterns in >90% of cells. While strong [*RNQ*+] cells also predominately displayed the p.f. pattern, the m.d. pattern was not observed. Interestingly, several of our [*RNQ*+] variants showed intermediate phenotypes on this spectrum. For instance, weak [*RNQ*+] and B1 W showed all three patterns with no dominant population easily recognizable, thereby highlighting the extent of conformational diversity present in our spontaneous [*RNQ*+] variants.

**Table 2:** Rnq1-GFP fluorescence patterns show diversity among [*RNQ*+] variants.

**[*RNQ*+] Variant**	**Rnq1-GFP Characterization**
[*rnq*-]	Diffuse fluorescence
s.d. low	One large focus (s.d.)
m.d. high	Multiple large foci (m.d.) and numerous petite foci (p.f.)
strong	Majority p.f.
medium	Majority s.d.
weak	Diversity: p.f., s.d., and m.d. present
very weak	Majority s.d., also m.d. present
A5	Majority s.d., also m.d. present
B1 P/W	Roughly 50% p.f., 50% s.d.
B1 W	Diversity: p.f., s.d., and m.d. present
C3	Majority s.d., also m.d. present
D3	Roughly 50% p.f., 50% s.d.
E1	Majority p.f., low percentage of s.d.
G4	Majority p.f., low percentage of s.d.
G6	Majority p.f., low percentage of s.d.

[*RNQ*+] variants expressing Rnq1(153-405)-GFP were characterized by the relative number of GFP aggregate-containing cells with single-dot (s.d.), multiple-dot (m.d.), or petite-foci (p.f.) fluorescence patterns. For each [*RNQ*+] variant, Z-stacks of at least 150 cells were analyzed.

### Spontaneous [*RN*Q+] variants propagate thermally stable amyloid structures

*In vitro* studies of Sup35 suggested that strong [*PSI*+] arises from amyloid fibers that have a relatively shorter protected core as compared to Sup35 aggregates of weak [*PSI*+] [40]. This difference in amyloid core length was found to positively correlate with fiber stability: the longer core of weak [*PSI*+] resulted in greater stability than strong [*PSI*+] [39]. Similarly, we found previously that more stable amyloid fibers of Rnq1-PFD produced weaker prion variants of [*RNQ*+], while less stable fibers propagated stronger [*RNQ*+] variants [43]. The resultant differences in phenotype with different [*PSI*+] and [*RNQ*+] variants were initially attributed to the ability of fibers to produce more free ends that are required for sequestering and converting monomeric protein [40]. Thus, less stable fibers produce more active “seeds” that convert soluble protein to the prion conformer faster, resulting in stronger biological phenotypes.

**Figure 4:**
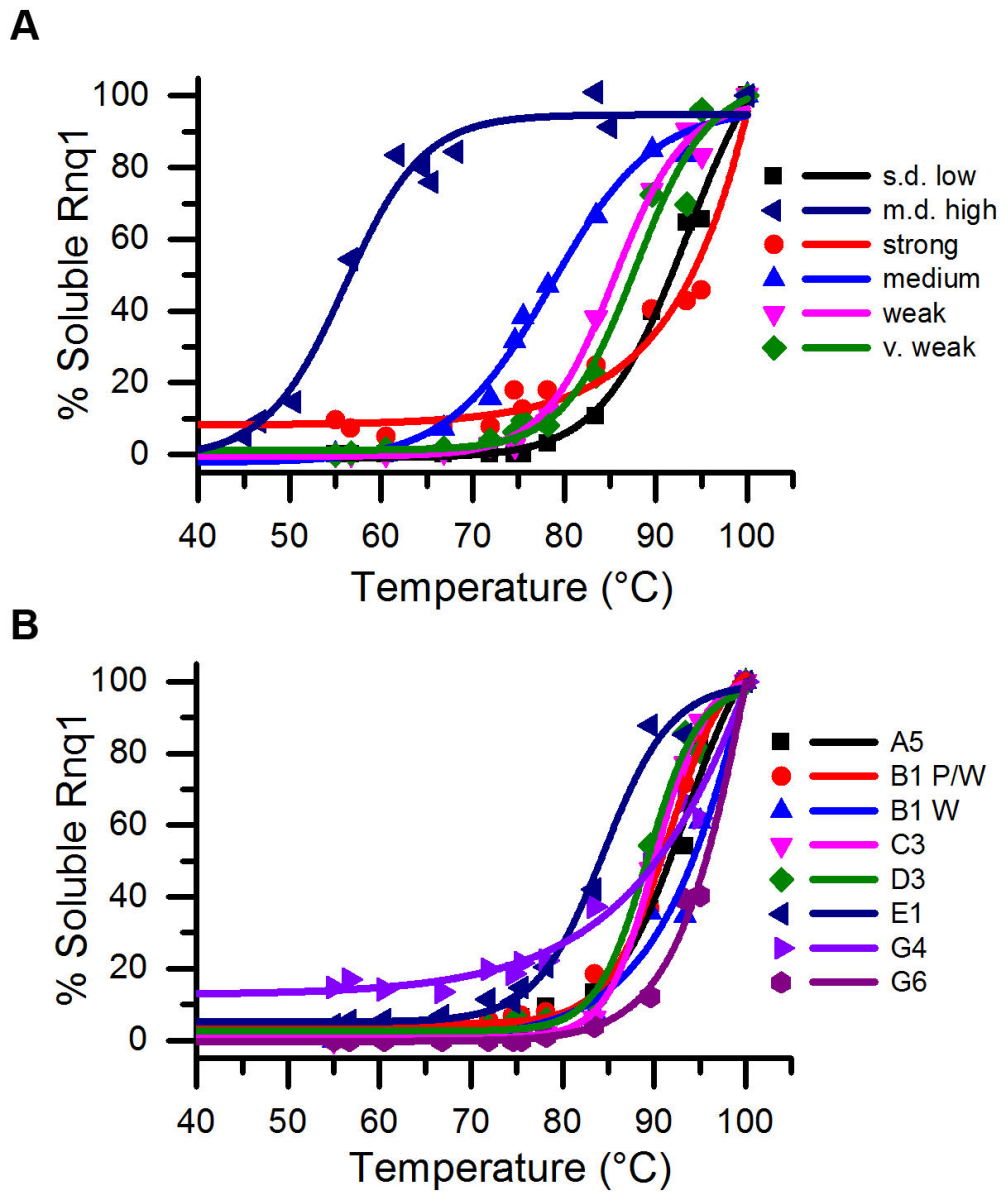
Aggregates of spontaneous [*RNQ*+] variants show similar thermal stability. Cell lysates were incubated for five minutes at different temperatures, run on an SDS-PAGE, and analyzed by western blot using an anti-Rnq1 antibody. The amount of soluble Rnq1 of both the (A) stable [*RNQ*+] variants and (B) unstable [*RNQ*+] variants was quantified with ImageJ and graphed as a percentage of soluble Rnq1 at 100°C. One representative experiment is shown, but in all cases, data were highly reproducible.

We were interested in determining whether the stability of our stable and unstable [*RNQ*+] variants would show a similar relationship with either their [*RRP*+] phenotype or efficiency in [*PSI*+] induction. Using thermal denaturation as an indicator of stability, we subjected cell lysates of our [*RNQ*+] variants to a temperature gradient and analyzed the samples by SDS-PAGE and western blot to determine the temperature at which 50% of the Rnq1 protein was liberated from the aggregates. Aggregates of all 12 [*RNQ*+] variants were shown to be very thermal stable with melting temperatures (T_m_) ranging from 80-95°C (Figure 4A-B). Rnq1 aggregates from medium [*RNQ*+] cells displayed the greatest sensitivity to temperature, with a T_m_ ~80°C, contrasting sharply with strong [*RNQ*+], in which Rnq1 aggregates remained largely intact at 90°C. While aggregates of s.d. low [*RNQ*+] showed a stability similar to our [*RNQ*+] variants, m.d. high [*RNQ*+] remained a striking outlier of all known [*RNQ*+] variants in exhibiting a T_m_ below 60°C, as demonstrated previously [54].

### SDD-AGE reveals variant-specific differences in aggregate size distribution

Previous studies with the [*PSI*+] and [*RNQ*+] prions have shown that the SDS-resistant, higher molecular weight protein aggregates can be resolved from the monomeric species and visualized using semi-denaturing detergent agarose gel electrophoresis (SDD-AGE) and western blot [37,54]. Furthermore, distinct prion variants can show different sizes of aggregates by SDD-AGE. For instance, many weak [*PSI*+] cell lysates harbor noticeably larger Sup35 aggregates than strong [PSI+] [37,39]. Slight differences also exist between some of the previously characterized [*RNQ*+] variants [54]. Therefore, we asked whether SDD-AGE analysis of our 12 stable and unstable [*RNQ+*] variants would reveal any additional differences in structural properties of the Rnq1 aggregates.

**Figure 5:**
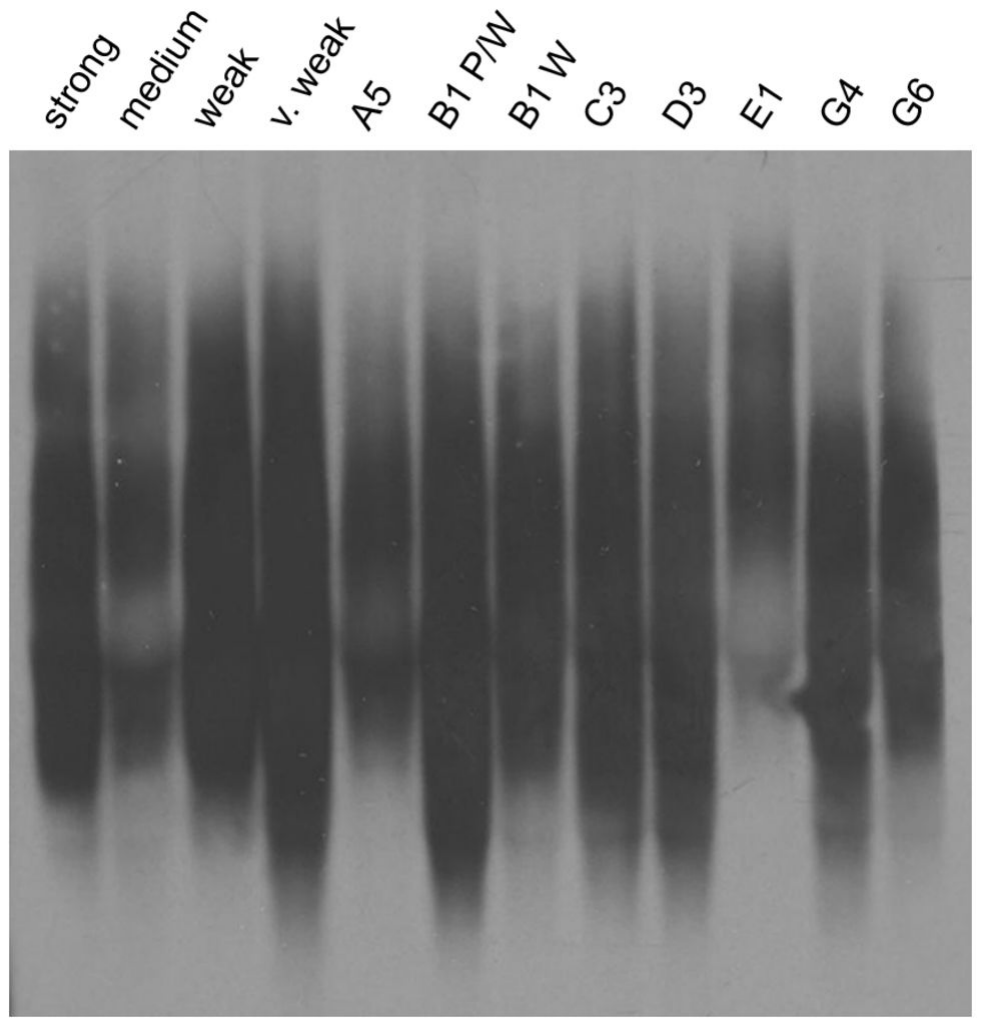
SDS-resistant aggregates of spontaneously formed [*RNQ*+] display modest, variant-specific differences in aggregate distribution. Aggregate distribution of [*RNQ*+] variants was analyzed using SDD-AGE by loading normalized amounts of protein from cell lysates onto an agarose gel containing SDS. Rnq1 was visualized by western blot using an anti-Rnq1 antibody.

Indeed, with cell lysates harboring each of our [*RNQ*+] variants, we found reproducible differences in aggregate distribution between many of the [*RNQ*+] variants of our subset (Figure 5). The higher molecular weight species of strong [*RNQ*+], medium [*RNQ*+], weak [*RNQ*+], B1 W, and G6 consistently migrated with a similar size as that of the previously published s.d. [*RNQ*+] variants (Figure 5 and data not shown). Other [*RNQ*+] variants, however, including very weak [*RNQ*+], C3, D3, and G4 contained a species that migrated slightly faster. A5 exhibited minor differences in aggregate distribution across multiple trials. Interestingly, B1 P/W and B1 W displayed modest, but reproducible differences as Rnq1 aggregates of B1 W were slightly larger than those found in B1 P/W lysates, despite having formed initially in the same colony. In striking contrast, E1 was the most unique and showed the bulk of the aggregated Rnq1 propagating in larger structures as compared to the other variants. Moreover, the aggregates of medium [*RNQ*+] consistently appeared much fainter in intensity, a property also observed with m.d. high [*RNQ*+] (data not shown).

## Discussion

In this study, we describe the isolation and characterization of several novel [*RNQ*+] variants that formed spontaneously *in vivo*. In the process of isolating these variants, we were able to estimate the rate of spontaneous [*RNQ*+] formation to be ~2.96x10^-6^. It was previously reported that the spontaneous rate of [*PSI*+] formation in [*psi*-] [*RNQ*+] yeast cells was 5.8x10^-7^ [63]. Hence, even when a high [*RNQ*+] variant is available to facilitate the formation of [*PSI*+], the *de novo* formation of [*RNQ*+] under our growth conditions occurs about 5 times more frequently. This places Rnq1 alongside other yeast prion proteins that all have a higher rate of formation than [*PSI*+], and are thought to have a functional role in the cell [69]. Moreover, while the ability to induce [*PSI*+] was not a requirement for isolation, every [*RNQ*+] variant analyzed in this study was shown to facilitate [*PSI*+] formation to some degree. These data suggest that the [*RNQ*+] prion, rather than simply acting as a promiscuous amyloid, might actually function to induce the formation of [*PSI*+] [70]. Although use of the RRP chimera remains the only way to easily measure the rate of [*RNQ*+] formation, one caveat here is that an extra copy of the Rnq1-PFD as part of RRP may contribute to a higher rate of spontaneous [*RNQ*+] formation.

In addition to being able to phenotypically monitor the formation of [*RNQ*+] with the use of RRP, the chimera also allowed us to identify distinct structures of aggregated Rnq1. After extensively characterizing the cellular and biochemical properties of 12 of the [*RNQ*+] variants that formed, we found that these different structures are indeed novel. While it is clear that no single property can fully differentiate between structural isomers of [*RNQ*+], when the properties we analyzed are considered collectively, each of our [*RNQ*+] variants displays a unique set of characteristics that distinguishes it from the others, as well as from the previously described [*RNQ*+] variants. 

Four of the [*RNQ*+] variants we isolated and characterized were mitotically stable, but differed in their [*RRP*+] phenotype. In contrast to [*PSI*+] variants and [*RNQ*+] variants formed from *in vitro* fibers that were previously reported [39,43], the degree of nonsense suppression of each of the stable [*RNQ*+] variants described in this study did not correlate to differences in stability or any of the other properties we examined.

Among the eight unstable [*RNQ*+] variants within our set, many displayed some degree of prion loss through mitotic division. While previous studies have documented a transient stage of mitotic instability in cells harboring newly induced [*PSI*+] and [RNQ+] [50,55], several of our [*RNQ*+] isolates were able to maintain their sectoring to red phenotypes through both restreaking and storage (data not shown). Strikingly, some of our unstable [*RNQ*+] variants exhibited more complex colony color phenotypes, such as A5, B1, D3, E1, and G6, in which colonies displayed sectors of various shades of pink. These findings suggest that multiple [*RNQ*+] variant types may have formed within the clonal population arising from a single cell. The B1 [*RNQ*+] variant epitomizes this observation, as our initial streak of cells harboring this prion variant produced white colonies with varying amounts of light pink sectoring, solid light pink colonies, and solid white colonies. Evidence of a single yeast strain harboring multiple prion conformations has also been observed with [*PSI*+]. Recently, clones isolated from a single [*PSI*+] colony exhibited a range of transmission profiles across an intra-species barrier, leading authors to propose that an ensemble, or cloud, of [*PSI*+] variants had been propagating in the parent cell [71]. Similarly, others have described an “unspecified” [*PSI*+] phenotype that was characterized by white colonies that sectored to pink, which gave rise to progeny carrying weak [*PSI*+], strong [*PSI*+], or unspecified [*PSI*+] [67]. In contrast to the “cloud” hypothesis, these authors consider an alternative possibility in which a single [*PSI*+] structure responsible for the unspecified [*PSI*+] phenotype is able to undergo a conformational maturation process into more than one distinct [*PSI*+] variant. Similarly, fibers of α-synuclein that were formed *in vitro* were recently shown to undergo a maturation process and form distinct aggregate conformations over time [53]. Both the “cloud” and “maturation” models may be applicable to our unstable [*RNQ*+] variants. Presumably, a host of factors, which also include competition between prion variants [51] and strain mutation [72], may play a role in dictating how prions propagate and which variants ultimately emerge phenotypically.

 The original set of [*RNQ*+] variants (referred to as [*PIN*+] variants) was obtained by over-expressing Sup35 in [*psi*-] [*rnq*-] cells and selecting for cells that converted to [*PSI*+] [51]. As *de novo* formation of [*PSI*+] requires [*RNQ*+] (which is the prion responsible for [*PIN*+]), [*RNQ*+] contemporaneously formed in these cells. In contrast, our [*RNQ*+] variants were isolated in a different manner using [*RRP*+]-mediated nonsense suppression, allowing us to obtain [*RNQ*+] variants with no connection to [*PSI*+] formation. Surprisingly, we found that all 40 of the spontaneous [*RNQ*+] variants induced [*PSI*+] at some level, albeit many facilitated [*PSI*+] formation at low to medium levels. These findings highlight the intrinsic relationship between [*RNQ*+] and [*PSI*+], and support the possibility that [*RNQ*+] functions primarily as a regulatory element in initiating the formation of [*PSI*+] [70].

 Many groups have speculated on the role of [*PSI*+] in nature, either as a harmful pathogenic state [73] or as a beneficial mechanism of generating heritable phenotypic diversity in response to stressful or shifting environmental conditions [74,75,76,77]. In the latter case, it is hypothesized that [*PSI*+]-mediated nonsense suppression allows translation of coding sequences downstream of stop codons, thereby creating novel gene products that may facilitate the evolution of new traits [76,78]. While reduced fidelity in translation termination could be deleterious, cells forming particular [*PSI*+] variants may in fact produce heritable, advantageous phenotypes that allow survival of the population without requiring immediate genetic change. Indeed, when cells were subjected to a range of environmental stresses, a correlation was found between the severity of stress and the frequency of [*PSI*+] formation, suggesting that cells may induce [*PSI*+] when a rapid adaptive response is needed [75]. With the discovery of [*PIN*+] as an essential factor for *de novo* [*PSI*+] formation, a two-prion system of epigenetic translation regulation was proposed [50]. The findings that we present here highlight the extent of conformational diversity that exists for the [*RNQ*+] prion. Such structural variation of [*RNQ*+] may contribute to differentially modulating the switch into a [*PSI*+] state, and influence the expansive phenotypic diversity observed with [*PSI*+]-dependent traits [76].

By isolating a number of novel [*RNQ*+] variants and adding to those previously described, we show the widespread variability in structures that the Rnq1 protein can assume. A high number of conformational possibilities have also been shown for [PSI+] [71,73], making the classification into strong and weak [*PSI*+] variants overly simplistic. We speculate that such diversity is not limited to these prion proteins, but may exist with many amyloidogenic proteins. Indeed, it was recently suggested that PrP may form over 30 distinct prion strains in humans [24]. Therefore, studying the numerous structural variants of [*RNQ*+] may help elucidate what biochemical properties or cellular factors contribute to the prion variant that propagates and how it manifests phenotypically.
